# Predicting immunoglobulin resistance in Kawasaki disease: an assessment of neutrophil to lymphocyte platelet ratio

**DOI:** 10.1186/s13052-022-01400-9

**Published:** 2022-12-30

**Authors:** Yuyao Lu, Yunjia Tang, Bo Wang, Xuan Li, Qiuqin Xu, Hui Chu, Haitao Lv, Meihua Lu, Yiming Qin

**Affiliations:** 1 Department of Cardiology, Children’s Hospital of Soochow University, No. 92, Zhongnan Street, Suzhou, P.R. China; 2 Department of Pediatrics, Changshu Hospital Affiliated to Nanjing University of Chinese Medicine, No. 6, Huanghe Road Changshu, P.R. China

**Keywords:** Neutrophil to lymphocyte platelet ratio, Intravenous immunoglobulin resistance, Kawasaki disease, Children

## Abstract

**Background:**

Kawasaki disease (KD) is an acute febrile illness of unknown etiology and predictors for intravenous immunoglobulin (IVIG) resistance have been widely explored in recent decades. Neutrophil to lymphocyte platelet ratio (NLPR) was reported to be associated with the outcomes in many diseases. However, its relationship with IVIG resistance has not be explored.

**Methods:**

The medical data of patients diagnosed with KD in Children’s Hospital of Soochow University between January 2019 and December 2020 were retrospectively reviewed and analyzed. Patients were trisected into three groups based on NLPR. Logistics regression was used to analyze the association between NLPR and IVIG resistance. Restricted cubic spine was used to exhibit the relationship. Sensitivity analysis and subgroup analysis were also carried out.

**Results:**

A total of 803 patients were included in the present study (61.8% males; median age: 24 months). IVIG resistance occurred in 74 (9.2%) patients. Multivariable-adjusted analyses revealed higher NLPR (odds ratio [95% confidence interval]: 1.12 [1.00-1.24]) was an independent predictor of IVIG resistance, which was strengthened by sensitivity analyses. The association of NLPR and IVIG resistance was not modified by age, sex, CALs, or days of IVIG initiation ≤ 4.

**Conclusion:**

NLPR may be a valuable prognostic marker in KD patients with IVIG resistance.

**Supplementary Information:**

The online version contains supplementary material available at 10.1186/s13052-022-01400-9.

## Background

Kawasaki disease (KD) is an acute febrile illness of unknown etiology, which predominantly affects children under 5 years of age. With a predilection for coronary artery lesions (CALs), it has become the most common cause of acquired heart diseases in developed countries [1]. Given that intravenous immunoglobulin (IVIG) resistance is one of the most important predictors for CALs, predictors for IVIG resistance have been widely explored and prediction models have been extensively established in recent decades [2–5].

Among them were neutrophil to lymphocyte ratio (NLR) and platelet to lymphocyte ratio (PLR) [6, 7]. Recognized as potential biomarkers of baseline inflammatory process, both two ratios were now widely reported in cardiovascular diseases [8, 9], neurological diseases [10, 11], and rheumatic diseases [12, 13], et al. The low-cost and consequent wide and easy availability in daily clinical practice has made them popular in clinical practice, with a higher ratio often indicating a poor prognosis.

More recently, neutrophil to lymphocyte platelet ratio (NLPR), which was obtained by either NLR and platelet counts or neutrophil counts and PLR, was reported to have a predictive value in disease prognosis [14, 15]. Considering that both neutrophils and platelets were indicative of inflammation, we hypothesized that NLPR was also associated with IVIG resistance in KD. However, no study has been carried out to investigate its value in KD. In the present study, we aimed to explore the association between NLPR and IVIG resistance.

## Methods

### Study population

We retrospectively reviewed and collected the medical data of patients diagnosed with KD in Children’s Hospital of Soochow University between January 2019 and December 2020. All patients were diagnosed based on American Heart Association (AHA) guidelines [1], and the first day of fever onset was defined as the first day of the disease. Patients were excluded if they: (1) had incomplete CALs status and NLPR value; (2) didn’t have IVIG treatment; (3) initiated IVIG in other hospitals. Patients were treated with a total dose of 2 g/kg of IVIG on diagnosis together with aspirin of 30–50 mg/kg/day in the acute stage, followed by 3–5 mg/kg/d aspirin after defervescence for at least 72 h. For patients with IVIG resistance, a second dose of IVIG, together with 2-4 mg/kg/day intravenous pulse methylprednisolone was administered.

### Data collection

Peripheral blood samples were routinely obtained within 24 h on admission and repeated 72 h after the administration of IVIG, or as appropriate. The laboratory data included white blood cell count (WBC) and its subpopulations, hematocrit, erythrocyte sedimentation rate (ESR), c-reactive protein (CRP), alanine aminotransferase (ALT), aspartate transaminase (AST), serum albumin, serum sodium, and serum potassium. If a laboratory indicator was performed two or more times before IVIG treatment, the results from the most recent IVIG were recorded. All patients received transthoracic echocardiography to record the status of the coronary arteries during the acute phase and were repeated before discharge and at 4 weeks, or as appropriate. The missing data are summarized in Additional file 1.

The study was reviewed and approved by the Ethics Committee of Children’s Hospital of Soochow University (No: 2020CS159). Informed consent was waived because of the retrospective nature of the study.

### Definitions

KD was diagnosed based on the 2017 AHA guidelines [1]. CALs were defined when Z-scores were larger than 2.5 based on the Dallaire equations [16], and (or) clear irregularity of the coronary artery intima. IVIG resistance was termed as a persistent or recrudescent fever ≥ 38.0℃ for more than 72 h after the initiation of IVIG [1]. NLPR was calculated as follows:

$$\mathrm{NLPR}=\frac{\mathrm{neutrophils}\;\mathrm{count}\;\left({\displaystyle\frac{{\mathrm x\;10}^9}{\mathrm L}}\right)\;\mathrm x\;100}{\mathrm{lymphocytes}\;\mathrm{count}\;\left({\displaystyle\frac{{\mathrm x\;10}^9}{\mathrm L}}\right)\;\mathrm x\;\mathrm{PLT}\;\left({\displaystyle\frac{{\mathrm x\;10}^9}{\mathrm L}}\right)}$$

### Statistical analyses

All statistical analyses in this study were performed using R (version 4.0.4). The data were presented as the mean ± standard deviation (SD) or median and quartiles for continuous variables and the comparisons were performed using one-way ANOVA or Kruskal–Wallis H test. Categorical variables were presented as numbers with percentages and were compared using chi-square or Fisher’s exact test. In the multivariable analyses, adjustments were made for potential confounders from the IVIG-resistant Kobayoshi’s prediction model [2] and from background knowledge such as age, sex, CRP, AST, ALT, serum albumin, and sodium, days of IVIG initiation ≤ 4. Percentages of neutrophils and platelet counts were not adjusted for the collinearity with NLPR. We excluded variables with incomplete data for more than 20%. Otherwise, we determined the missing data by multiple imputation methods. NLPR was trisected in T1 (< 33rd percentile) T2 (33rd − 67th percentile), T3 (≥ 67th percentile), and were compared. Area under receiver operating characteristic (ROC) curve (AUC) was also calculated. A two-sided *P* < 0.05 was considered statistically significant.

We additionally carried out sensitivity analyses. First, we grouped NLPR into < 20th percentile (T1), 20th − 80th percentile (T2), and ≥ 80th percentile (T3) and did the multivariable logistic regression again. Second, we used variables in the present study and selected the confounders. Variables with *P* < 0.20 in the univariate analysis were included in the multivariable analysis.

Given that there are two crossovers of peripheral neutrophils and lymphocytes in childhood, which occur between 4 ~ 6 days after birth and 4 ~ 6 years old, additional interaction analyses were performed to determine possible effect modification by the subgroups. We grouped all participants into < 48 months and ≥ 48 months since that KD scarcely occurred in neonates. We didn’t group patients ≥ 48 months in more groups because there were only 38 patients ≥ 72 months. Besides, interaction analyses for sex, CALs, delayed IVIG treatment, and days of IVIG initiation ≤ 4 were also carried out.

## Results

### Patient characteristics

**Fig. 1:**
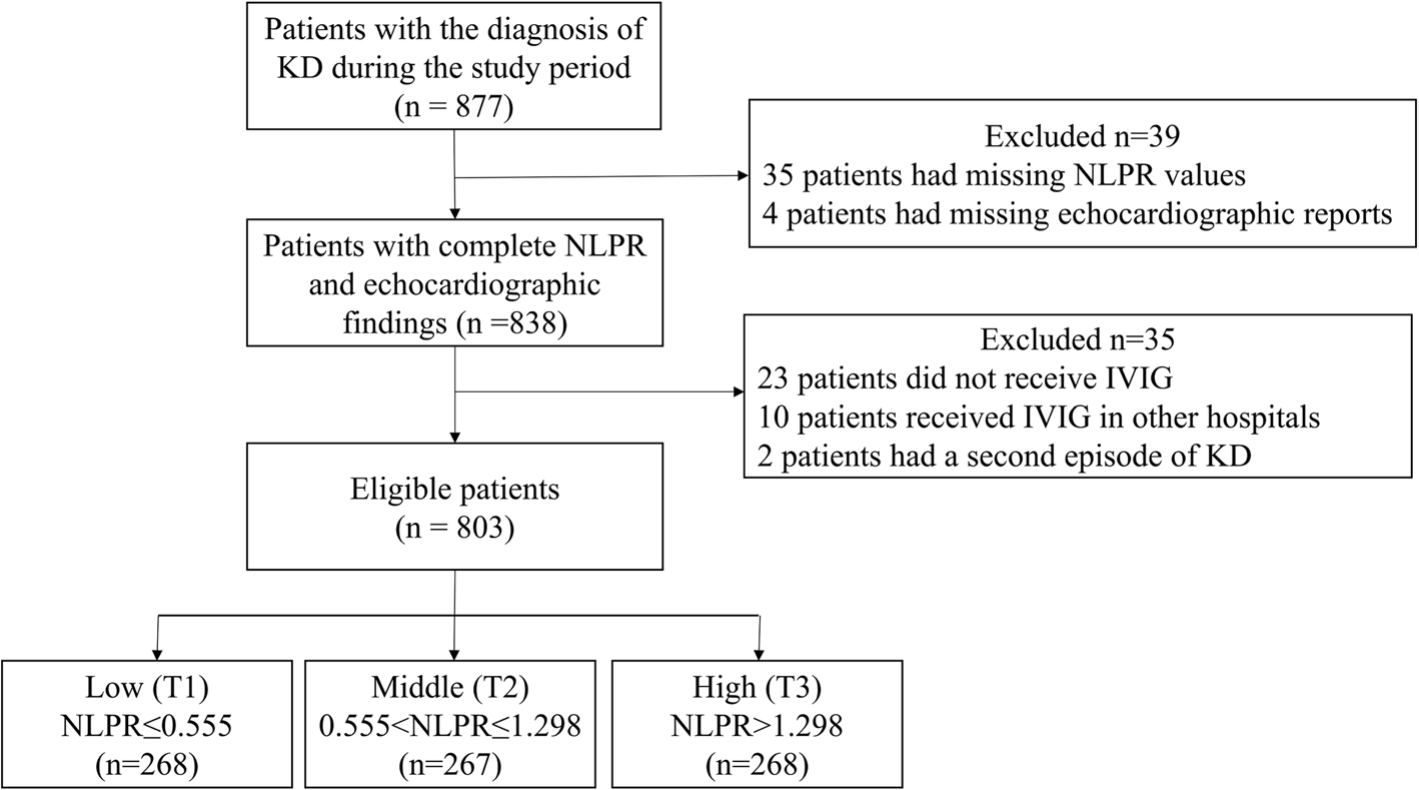
Flow diagram of the study. KD: Kawasaki disease; NLPR: neutrophil to lymphocyte platelet ratio; IVIG: intravenous immunoglobulin

A total of 803 patients were included in the final analysis (Fig. 1), including 442 (61.8%) males and 273 (38.2%) females, with a male-to-female ratio of 1.62. The median age was 24 months. As shown in Table 1, patients in T3 were older, less likely to be IKD, and less likely to have delayed IVIG initiation. Besides, they had higher levels of CRP, ESR, hematocrit, AST, ALT, and lower levels of albumin, serum sodium, and potassium (*P* < 0.01). Moreover, patients in T3 tended to have longer days of hospitalization. 74 (9.2%) had IVIG resistance. Notably, patients in T3 had a higher rate of IVIG resistance than those in T1 and T2 (14.9% in T3, 6.7% in T2, and 6.0% in T1, respectively, see Table 2. However, no significant difference in CALs was found among the three groups (*P* = 0.159).

**Table 1:** Patient characteristics

	level	T1	T2	T3	*P* value
n		268	267	268	
Sex	Female	117 (43.7)	110 (41.2)	119 (44.4)	0.736
	Male	151 (56.3)	157 (58.8)	149 (55.6)	
Age (months)		15.00 [10.00, 25.25]	24.00 [15.00, 36.00]	37.00 [24.00, 55.00]	< 0.001
Age < 1 year	No	183 (68.3)	222 (83.1)	252 (94.0)	< 0.001
	Yes	85 (31.7)	45 (16.9)	16 ( 6.0)	
Days of IVIG initiation < 5	No	249 (92.9)	250 (93.6)	249 (92.9)	0.93
	Yes	19 (7.1)	17 (6.4)	19 (7.1)	
Days of IVIG initiation ≥ 10	No	217 (81.0)	235 (88.0)	255 (95.1)	< 0.001
	Yes	51 (19.0)	32 (12.0)	13 ( 4.9)	
Incomplete KD	No	198 (73.9)	220 (82.4)	234 (87.3)	< 0.001
	Yes	70 (26.1)	47 (17.6)	34 (12.7)	
CRP (mg/dL)		48.24 [26.80, 76.09]	64.47 [38.58, 96.48]	86.35 [57.21, 122.80]	< 0.001
ESR (mm/h)		33.00 [18.00, 46.00]	35.00 [22.00, 54.00]	42.00 [25.00, 56.00]	< 0.001
NLPR		0.35 [0.23, 0.45]	0.86 [0.67, 1.04]	2.16 [1.65, 3.30]	< 0.001
Hematocrit (%)		0.33 (0.03)	0.34 (0.03)	0.34 (0.03)	0.006
Albumin (g/L)		39.89 (3.31)	40.27 (5.52)	38.88 (4.48)	0.001
AST (U/L)		31.95 [24.95, 45.52]	32.30 [25.45, 43.65]	38.95 [27.08, 80.15]	< 0.001
ALT (U/L)		20.65 [12.95, 42.55]	22.50 [13.40, 54.90]	37.10 [13.88, 134.08]	< 0.001
Sodium (mmol/L)		136.38 (2.45)	135.42 (2.45)	133.93 (3.30)	< 0.001
Potassium (mmol/L)		4.11 (0.49)	3.99 (0.50)	3.80 (0.50)	< 0.001

Data are presented as mean ± standard deviation or median and quartiles or numbers with percentages

*IVIG* Intravenous immunoglobulin, *KD* Kawasaki disease, *CRP* C-reaction protein, *ESR* Erythrocyte sedimentation rate, *NLPR* Neutrophil to lymphocyte platelet ratio, *AST* Aspartate transaminase, *ALT* Alanine aminotransferase

**Table 2:** Outcomes of the study patients

	level	T1	T2	T3	Total	*P*
n		268	267	268	803	
IVIG resistance	No	252 (94.0)	249 (93.3)	228 (85.1)	729 (90.8)	< 0.001
	Yes	16 (6.0)	18 (6.7)	40 (14.9)	74 (9.2)	
Days of hospitalization		10.49 (4.71)	10.44 (4.53)	11.75 (4.18)	10.89 (4.52)	0.001
Days of fever duration		7.28 (2.39)	7.18 (2.29)	7.26 (1.86)	7.24 (2.19)	0.841
Coronary artery lesions	No	187 (69.8)	191 (71.5)	206 (76.9)	584 (72.7)	0.159
	Yes	81 (30.2)	76 (28.5)	62 (23.1)	219 (27.3)	

Data are presented as mean ± standard deviation or numbers with percentages

*IVIG* Intravenous immunoglobulin

### Relationship between NLPR and IVIG resistance

**Fig. 2:**
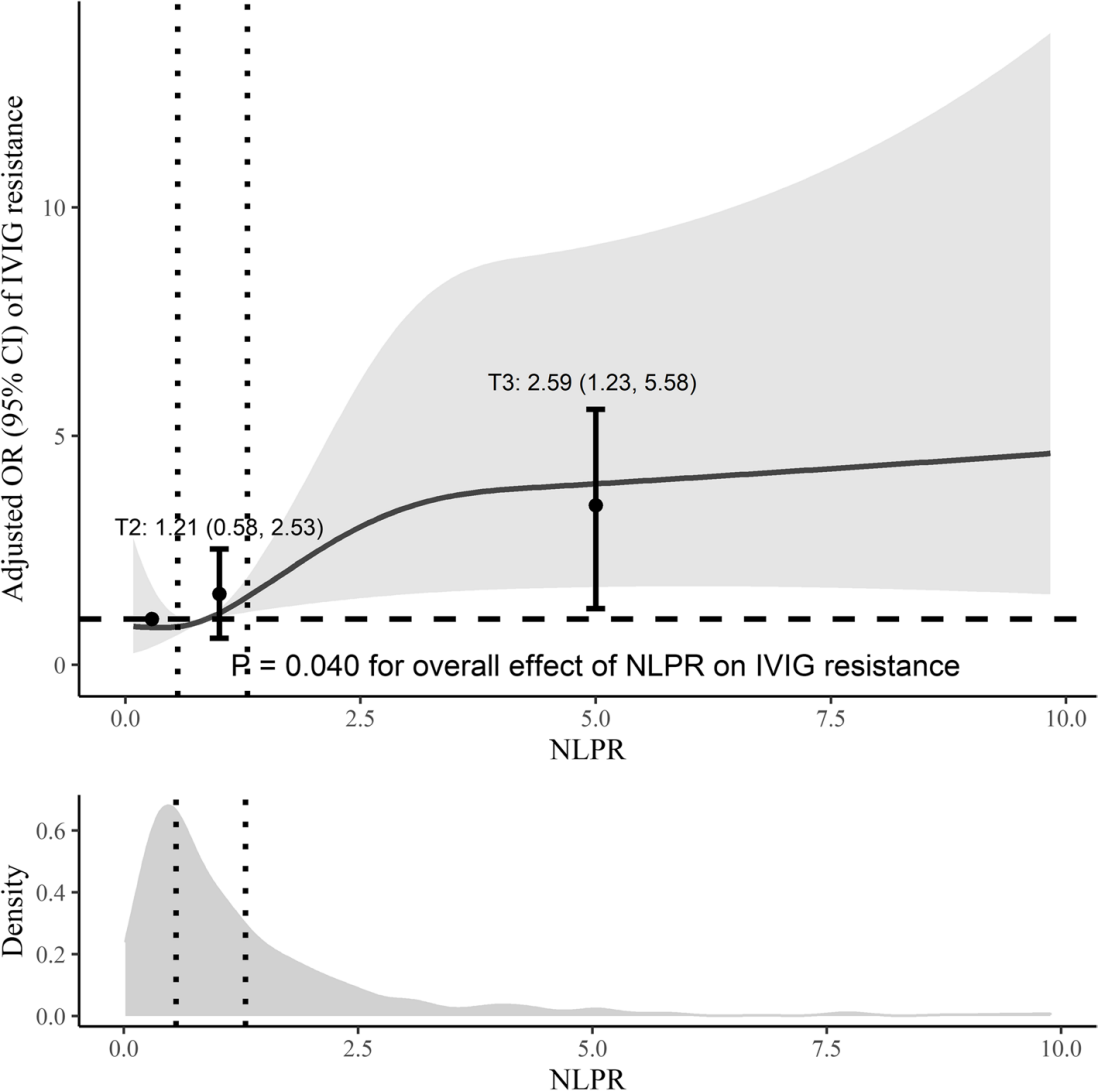
The adjusted odds ratio (OR) for NLPR for IVIG resistance. The solid line indicates the odds ratio of NLPR. The shaded area represents the 95% confidence interval (CI). Dots indicate the OR ratio according to each group. Error bars represent the 95% CI. The vertical dotted lines trisected patients. NLPR: neutrophil to lymphocyte platelet ratio; IVIG: intravenous immunoglobulin

**Fig. 3:**
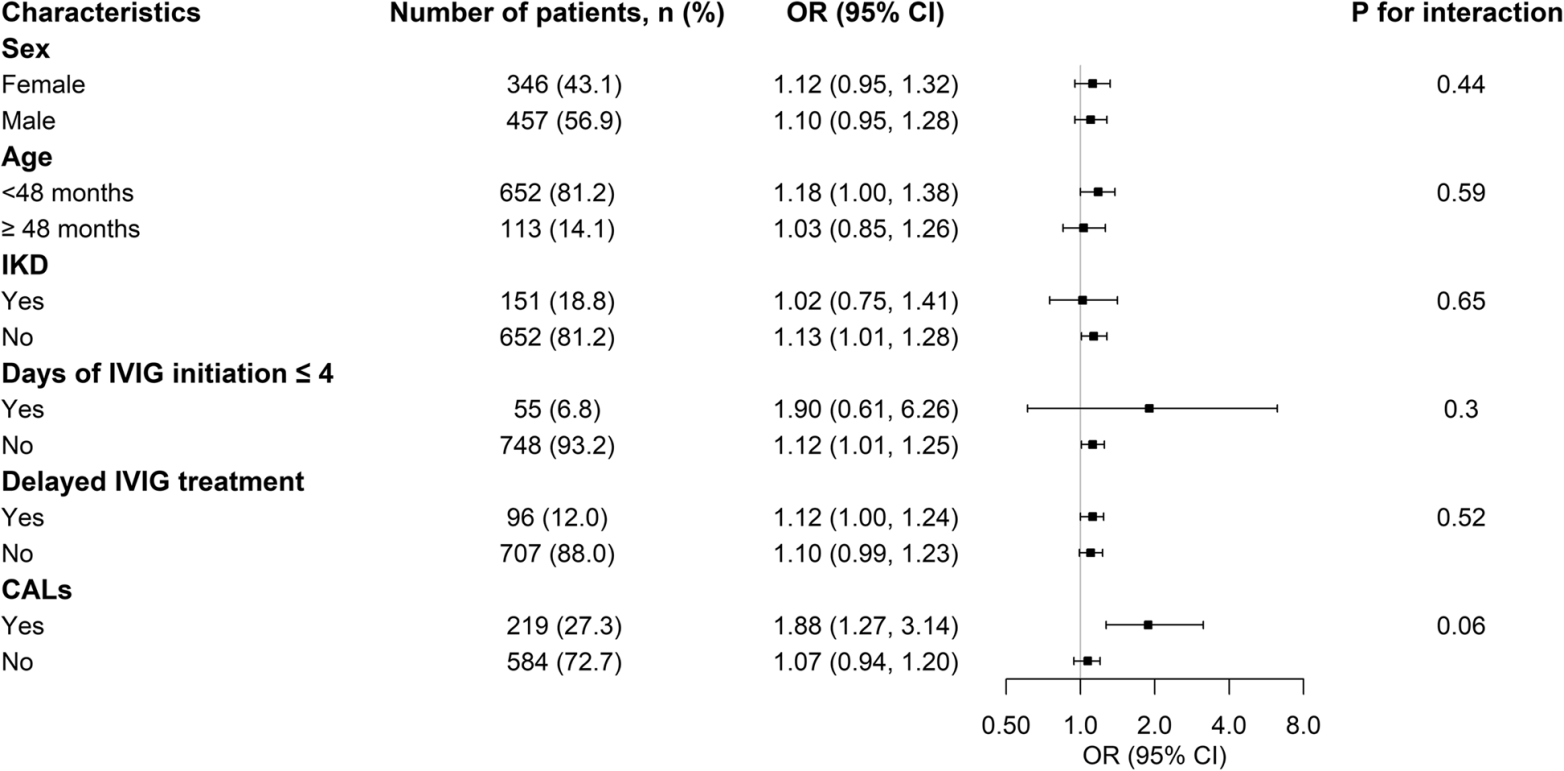
Subgroup analysis. IVIG: intravenous immunoglobulin; CALs: coronary artery lesions; OR: odds ratio; CI: confidence interval; IKD: incomplete Kawasaki disease

The results of the logistic analysis of IVIG resistance are shown in Table 3. In the univariate analysis, NLPR as a continuous variable was negatively associated with IVIG resistance (*P* = 0.001). Even after adjusting the confounders, the difference remained significant (*P* = 0.040). When NLPR was trisected, patients with middle NLPR (T2) and high NLPR (T3) were associated with a higher incidence of IVIG resistance. However, the difference in T2 was insignificant (odds ratio [95% confidence interval]: 1.21 [0.58, 2.53]). T3 had a higher incidence of IVIG resistance in both the crude and multivariable-adjusted models (odds ratio [95% confidence interval]: 2.76 [1.53, 5.20] and 2.59 [1.23, 5.58], respectively). P for trend in both two models were statistically significant (*P* < 0.05). NLPR was determined to be independently associated with IVIG resistance (Fig. 2). Subgroup analysis showed age, sex, CALs, IKD, delayed IVIG treatment, and days of IVIG initiation ≤ 4 did not modify the association between NLPR and IVIG resistance (Fig. 3).

**Table 3:** Impact of NLPR on IVIG resistance

	IVIG resistance	Unadjusted	Multiviable adjusted^a^
	No	OR (95% CI) P	OR (95% CI) P
NLPR	74/803	1.14 (1.05, 1.24) 0.001	1.12 (1.00, 1.24) 0.040
T1 (< 0.555)	16/268	1.0 (reference)	1.0 (reference)
T2 (0.555–1.298)	18/267	1.14 (0.57, 2.30) 0.714	1.21 (0.58, 2.53) 0.611
T3 (≥ 1.298)	40/268	2.76 (1.53, 5.20) 0.001	2.59 (1.23, 5.58) 0.013
P for trend	< 0.001	< 0.001	0.010

*NLPR* Neutrophil to lymphocyte platelet ratio, *IVIG* Intravenous immunoglobulin, *CI* Confidence interval, *OR* Odds ratio

^a^ Adjusted for age, sex, c-reaction protein, aspartate transaminase, alanine aminotransferase, serum albumin, sodium, and days of IVIG initiation ≤ 4

## Sensitivity analyses

Our findings were preserved across the sensitivity analyses (Table 4). NLPR as a continuous variable and as a categorical variable were both independently associated with IVIG resistance (*P* < 0.05). The comparisons of variables in patients with and without IVIG resistance are shown in Additional file 2. The logistic analyses of IVIG resistance using data in the present study are shown in Additional file 3. Age, serum albumin, NLPR, and days of IVIG initiation ≤ 5 were selected as independent predictors of IVIG resistance.

**Table 4:** Sensitivity analysis

	Sensitivity analysis I^a^	Sensitivity analysis II^b^
	OR (95% CI) P	OR (95% CI) P
NLPR	1.12 (1.00, 1.24) 0.040	1.17 (1.07, 1.29) < 0.001
T1	1.0 (reference)	1.0 (reference)
T2	1.11 (0.52, 2.53) 0.800	1.367 (0.67, 2.82) 0.389
T3	3.92 (1.55, 10.44) 0.005	3.55 (1.84, 7.11) < 0.001
P for trend	0.001	< 0.001

*NLPR* Neutrophil to lymphocyte platelet ratio, *IVIG* Intravenous immunoglobulin, *CI* Confidence interval, *OR* Odds ratio

^a^ Adjusted for age, sex, c-reaction protein, aspartate transaminase, alanine aminotransferase, serum albumin, sodium, and days of IVIG initiation ≤ 4. NLPR levels were re-categorized into < 20th percentile (T1), 20th − 80th percentile (T2), and > 80th percentile (T3)

^b^ Adjusted for age, serum albumin, and days of IVIG initiation ≤ 5

### ROC analysis

AUC of NLPR was 0.638 (95% confidence interval: [0.567–0.709]) with a sensitivity of 46% and a specificity of 81%. The cut-off value of NLPR was 1.8. The prediction model including NLPR, age, serum albumin, and days of IVIG initiation ≤ 4 had an AUC of 0.697 (95% confidence interval: [0.633–0.761]), with a sensitivity of 71% and a specificity of 64%.

## Discussion

The retrospective study of 803 KD patients in the present study showed that a higher level of NLPR was associated with an increased incidence of IVIG resistance. Furthermore, the relationship was not modified by age, sex, CALs, IKD, or days of IVIG initiation.

The positive correlation between NLPR and IVIG resistance was in consistent with what we expected in clinical practice as elevated neutrophils and reduced platelet counts were both considered predictors of IVIG resistance in previous studies [2, 4, 5, 17]. On the other hand, a low level of lymphocyte count was also identified as an independent predictor in a recent study [17]. However, all these prediction models assessed neutrophils, lymphocytes, and platelet counts separately. Accordingly, percentages or absolute cell counts were used in each study with no unanimous standards.

NLPR, which was calculated by neutrophils, lymphocytes, and platelet counts, partly reflected the potential roles of all these variables simultaneously. Neutrophils are the most abundant type of white blood cells in peripheral blood and play crucial roles in inflammation and infection. A sudden increase in neutrophils led to increased oxygen intermediate, neutrophil elastase, and myeloperoxidase in the acute stage of KD [18–20], which on one hand caused self-destruction and on the other hand, reflected disease severity. Our results showed that the absolute count of lymphocytes and percentage of lymphocytes were both significantly lower in patients with IVIG resistance, which was in line with previous studies [17, 21]. The decrease in lymphocytes was a consequence of dynamically changed cell abundances in the acute stage of KD, with B cells decreasing and T cells varying among different subgroups [22].

Previous studies had pointed out that neutrophils infiltrated both the myocardium and the coronary arteries of the autopsied KD patients [23, 24], indicating a potential role of neutrophils in CALs and myocarditis. However, higher NLPR was not recognized as a predictor of CALs in the present study. Prediction of CALs, together with IVIG resistance was the issue of major interest in KD. However, although CALs were the consequence of disease severity and IVIG resistance was a form of disease severity, the risk factors for the two were not quite the same. The potential reasons still lay in the pathogenesis of KD and remained to be explored.

Of note, we found that patients with higher NLPR tended to have a more severe disease course, which was indicated by other laboratory variables, such as higher levels of CRP, ESR, AST, ALT, and lower levels of serum albumin, serum sodium, and potassium, almost all of which were referred to IVIG resistance in previous studies [1–5]. Besides, patients with higher NLPR were older and less likely to have delayed treatment, which could be explained by lymphocyte dominance in white blood cells in healthy children under 4–6 years old and a surge of platelets in the later stage of the disease course [25]. However, neither age nor days of IVIG initiation modified the relationship between NLPR and IVIG resistance. The result remained the same regarding sex, CALs, and IKD.

There were some limitations in the present study. First, selection biases existed due to the retrospective nature of the study. Second, numbers of prediction models had been established all over the world, with none of them having excellent performance in all populations [26–28]. The reasons lay in the methods to establish the models, the variables included in the analysis, the different genetic backgrounds of the study population, et al. Here we identified NLPR, age, serum albumin, and days of IVIG initiation ≤ 4 as predictors of IVIG resistance in the study population. Unfortunately, the performance of the prediction model was not excellent with regards to its AUC of 0.697. Further studies are warranted to explore a more accurate and applicable model in this area.

## Conclusion

NLPR may be a valuable prognostic marker in KD patients with IVIG resistance.

## Supplementary Information

**Additional file 1.** Missing values. Shows the missing data in laboratory variables.

**Additional file 2.** Comparisons of variables in patients with and without IVIG resistance. Shows the comparisons of variables in patients with and without IVIGresistance.

**Additional file 3.** Logistic analysis of IVIG resistance. Shows the univariate and multivariable logistic analyses of IVIGresistance using the present dataset

## Data Availability

The datasets used and/or analyzed during the current study are available from the corresponding author on reasonable request.

## Abbreviations

NLPR: Neutrophil to lymphocyte platelet ratio
IVIG: Intravenous immunoglobulin
KD: Kawasaki disease
CALs: Coronary artery lesions
NLR: Neutrophil to lymphocyte ratio
PLR: Platelet to lymphocyte ratio
AHA: American Heart Association
WBC: White blood cell
ESR: Erythrocyte sedimentation rate
CRP: C-reaction protein
ALT: Alanine aminotransferase
AST: Aspartate transaminase
SD: Standard deviation
CI: Confidence interval
ROC: Receiver operating characteristic
AUC: Area under the curve
