# Supplementary material for: Predicting immunoglobulin resistance in Kawasaki disease: an assessment of neutrophil to lymphocyte platelet ratio
Source: Ital J Pediatr. 2022 Dec 30;48:208. doi: 10.1186/s13052-022-01400-9 (PMC9805255; doi:10.1186/s13052-022-01400-9)
Supplement: Supplementary file 1 — Additional file 1. Missing values. Shows the missing data in laboratory variables. [file 13052_2022_1400_MOESM1_ESM.docx]

Additional file 1. Missing values

| Variables | Number, n (%) |
| --- | --- |
| Erythrocyte sedimentation rate | 87 (10.8) |
| Hematocrit | 2 (0.2) |
| Surum albumin | 4 (0.4) |
| Alanine aminotransferase | 4 (0.4) |
| Aspartate transaminase | 4 (0.4) |
| Troponin T | 230 (28.6) |
| Sodium | 22 (2.7) |
| Potassium | 22 (2.7) |
