# Supplementary material for: Predicting immunoglobulin resistance in Kawasaki disease: an assessment of neutrophil to lymphocyte platelet ratio
Source: Ital J Pediatr. 2022 Dec 30;48:208. doi: 10.1186/s13052-022-01400-9 (PMC9805255; doi:10.1186/s13052-022-01400-9)
Supplement: Supplementary file 2 — Additional file 2. Comparisons of variables in patients with and without IVIG resistance. Shows the comparisons of variables in patients with and without IVIGresistance. [file 13052_2022_1400_MOESM2_ESM.docx]

Additional file 2. Comparisons of variables in patients with and without IVIG resistance

|  | Level | Non-IVIG resistance | IVIG resistance | | P |
| --- | --- | --- | --- | --- | --- |
| n |  | 729 | 74 | |  |
| Sex (%) | Female | 320 (43.9) | 26 (35.1) | | 0.185 |
|  | Male | 409 (56.1) | | 48 (64.9) | |
| Age | | 25.00 [13.00, 41.00] | 20.50 [14.00, 35.00] | | 0.193 |
| Days of IVIG initiation < 5 | No | 684 (93.8) | 64 (86.5) | | 0.032 |
|  | Yes | 45 ( 6.2) | | 10 (13.5) | |
| Days of IVIG initiation ≥ 10 | No | 639 (87.7) | 68 (91.9) | | 0.377 |
|  | Yes | 90 (12.3) | | 6 ( 8.1) | |
| Incomplete KD (%) | No | 589 (80.8) | 63 (85.1) | | 0.451 |
|  | Yes | 140 (19.2) | | 11 (14.9) | |
| CRP (mg/dL) | | 63.68 [38.28, 97.74] | 90.13 [41.55, 133.74] | | 0.005 |
| ESR (mm/h) | | 35.00 [21.00, 54.00] | 37.50 [17.25, 51.50] | | 0.781 |
| Platelet counts (×10^9^/L) | | 350.42 (119.59) | 308.76 (111.56) | | 0.004 |
| Percentage of lymphocytes (%) | | 24.65 [16.72, 33.27] | 17.90 [10.60, 30.85] | | 0.001 |
| Percentage of neutrophils (%) | | 64.76 (15.37) | 70.72 (16.12) | | 0.002 |
| Neutrophil counts (×10^9^/L) | | 8.91 [6.55, 12.41] | 10.10 [6.21, 12.51] | | 0.654 |
| Lymphocyte counts (×10^9^/L) | | 3.35 [2.17, 4.82] | 2.53 [1.41, 4.19] | | 0.002 |
| NLPR | | 0.82 [0.44, 1.57] | 1.45 [0.61, 2.58] | | <0.001 |
| Hematocrit (%) | | 0.34 (0.03) | 0.33 (0.04) | | 0.023 |
| Albumin (g/L) | | 39.88 (4.33) | 37.68 (6.10) | | <0.001 |
| AST (U/L) | | 33.20 [25.40, 49.30] | 39.70 [31.95, 59.85] | | 0.003 |
| ALT (U/L) | | 23.30 [13.30, 65.70] | 33.05 [16.20, 116.82] | | 0.018 |
| Sodium (mmol/L) | | 135.37 (2.84) | 133.96 (3.52) | | <0.001 |
| Potassium (mmol/L) | | 3.98 (0.50) | 3.87 (0.58) | | 0.085 |

Data are presented as mean ± standard deviation or median and quartiles or numbers with percentages.

IVIG: intravenous immunoglobulin, KD: Kawasaki disease, CRP: c-reaction protein, ESR: erythrocyte sedimentation rate, NLPR: neutrophil to lymphocyte platelet ratio, AST: aspartate transaminase, ALT: alanine aminotransferase.
