# Supplementary material for: Predicting immunoglobulin resistance in Kawasaki disease: an assessment of neutrophil to lymphocyte platelet ratio
Source: Ital J Pediatr. 2022 Dec 30;48:208. doi: 10.1186/s13052-022-01400-9 (PMC9805255; doi:10.1186/s13052-022-01400-9)
Supplement: Supplementary file 3 — Additional file 3. Logistic analysis of IVIG resistance. Shows the univariate and multivariable logistic analyses of IVIGresistance using the present dataset [file 13052_2022_1400_MOESM3_ESM.docx]

Additional file 3. Logistic analysis of IVIG resistance

|  | Levels | OR (95% CI) | |
| --- | --- | --- | --- |
|  |  | Univariate | Multivariable |
| Sex | Female | 1.0 | - |
|  | Male | 1.44 (0.88, 2.38) |  |
| Age (months) |  | 0.99 (0.98, 1) | 0.98 (0.97,1) |
| Days of IVIG initiation < 5 | No | 1.0 |  |
|  | Yes | 2.37 (1.14, 4.94) | 3.01 (1.39,6.51) |
| CRP (mg/dL) |  | 1.01 (1.00, 1.01) | - |
| NLPR |  | 1.14 (1.05, 1.24) | 1.11 (1,1.24) |
| Hematocrit (%) |  | 0 (0, 0.33) | - |
| Albumin (g/L) |  | 0.89 (0.84, 0.94) | 0.92 (0.86,0.98) |
| AST (U/L) |  | 1.00 (1.00, 1.00) | - |
| ALT (U/L) |  | 1.00 (1.00, 1.00) | - |
| Sodium (mmol/L) |  | 0.86 (0.8, 0.93) | - |
| Potassium (mmol/L) |  | 0.65 (0.41, 1.06) | - |

IVIG: intravenous immunoglobulin, KD: Kawasaki disease, CRP: c-reaction protein, ESR: erythrocyte sedimentation rate, NLPR: neutrophil to lymphocyte platelet ratio, AST: aspartate transaminase, ALT: alanine aminotransferase.
